# Quantitative Assessment of Hand Function in Healthy Subjects and Post-Stroke Patients with the Action Research Arm Test

**DOI:** 10.3390/s22103604

**Published:** 2022-05-10

**Authors:** Jesus Fernando Padilla-Magaña, Esteban Peña-Pitarch, Isahi Sánchez-Suarez, Neus Ticó-Falguera

**Affiliations:** 1 Escola Politècnica Superior d’Enginyeria de Manresa (EPSEM), Polytechnic University of Catalonia (UPC), 08242 Manresa, Spain; esteban.pena@upc.edu; 2 Department of Manufacturing Technologies, Polytechnic University of Uruapan Michoacán, Uruapan 60210, Michoacán, Mexico; i.sanchez@upu.edu.mx; 3 Physical Medicine and Rehabilitation Service, Althaia Xarxa Assistencial de Manresa, 08243 Manresa, Spain; nico@althaia.cat

**Keywords:** hand, stroke, rehabilitation, finger joints, data glove

## Abstract

The Action Research Arm Test (ARAT) can provide subjective results due to the difficulty assessing abnormal patterns in stroke patients. The aim of this study was to identify joint impairments and compensatory grasping strategies in stroke patients with left (LH) and right (RH) hemiparesis. An experimental study was carried out with 12 patients six months after a stroke (three women and nine men, mean age: 65.2 ± 9.3 years), and 25 healthy subjects (14 women and 11 men, mean age: 40.2 ± 18.1 years. The subjects were evaluated during the performance of the ARAT using a data glove. Stroke patients with LH and RH showed significantly lower flexion angles in the MCP joints of the Index and Middle fingers than the Control group. However, RH patients showed larger flexion angles in the proximal interphalangeal (PIP) joints of the Index, Middle, Ring, and Little fingers. In contrast, LH patients showed larger flexion angles in the PIP joints of the Middle and Little fingers. Therefore, the results showed that RH and LH patients used compensatory strategies involving increased flexion at the PIP joints for decreased flexion in the MCP joints. The integration of a data glove during the performance of the ARAT allows the detection of finger joint impairments in stroke patients that are not visible from ARAT scores. Therefore, the results presented are of clinical relevance.

## 1. Introduction

Stroke remains the second-leading cause of death and the third-leading cause of death and disability combined globally. Projections show that the burden of stroke will not decrease in the next decade or beyond [1]. An important contributing factor is that the number of older persons in Europe is rising, with a projected increase of 35% between 2017 and 2050 [2]. Stroke is caused by the death of brain cells as a result of blockage of a blood vessel supplying the brain (ischemic stroke) or bleeding into or around the brain (hemorrhagic stroke) [3]; the disability and the rehabilitation that is needed post-stroke depends on the size of the brain injury and the particular brain circuits that are damaged [4]. The most common sequelae caused by stroke is motor impairment, which impairs function in muscle movement or mobility [5]. One of the most affected parts are the upper extremities (UEs) of the human body; movement problems in these parts limit the quality of life by limiting the ability to perform activities of daily living (ADLs). The hand is one of the essential tools of the human body, allowing us to perform a wide variety of actions to interact with the environment, such as touching, reaching, holding, grasping, and manipulating different types of objects. People who suffer the loss of mobility in the hand endure a tremendous negative impact on their living standards, causing problems in their family, work, and social environment. Therefore, the rehabilitation process after a stroke is fundamental to prevent deterioration of function, reduce motor disability and reintegrate patients into their ADLs [6]. Stroke rehabilitation is divided into three phases: acute phase (first week), subacute phase (one–six months), and chronic phase (after six months) [7]. In order to evaluate the patient’s progress during the rehabilitation program, it is highly recommended to use standardized outcome measures (OMs) with good psychometric properties. There is a wide range of upper extremity rehabilitation OMs (e.g., motor function, muscle strength, dexterity, global stroke severity, and others) [8]. Many physical therapists have assessed the upper limb function in post-stroke patients with the Action Research Arm Test (ARAT). The ARAT is a measurement tool used to assess UE functional limitations. The test described by Lyle [9] evaluates 19 tests of arm motor function that assess a patient’s ability to handle objects differing in size, weight, and shape. Each test is given an ordinal score of 0, 1, 2, or 3, with higher values indicating better arm motor status [10]. The test has shown good reliability and validity [10,11,12]. The ARAT, like other OMs, is evaluated by an examiner who determines the score of each test. The scoring process can lead to subjective results due to the difficulty of assessing abnormal patterns in patients after stroke.

Therefore, as a result of technological advances, wearable sensors have been incorporated during the performance of various OMs in several clinical investigations. The use of sensors allows having more quantitative and sensitive assessment methods during clinical rehabilitation of the UEs. Most research studies have used inertial measurement units (IMUs) while performing the ARAT. Carpinella et al. proposed a method to discriminate between healthy subjects and multiple sclerosis patients wearing a single inertial sensor on the wrist [13]. Nam et al. obtained a database of the workspace and range of motion (ROM) of the major joints of the UEs in healthy subjects using a wearable motion capture system based on an (IMU) [14]. Repnik et al. proposed a system of IMUs for kinematic quantification and electromyography (EMG) sensors for muscle activity analysis in stroke patients [15]. Held et al. measured arm kinematics in stroke patients during different stages of the rehabilitation process using an Xsens full-body motion capture suit (Xsens Technologies, Enschede, Netherlands) [16]. In contrast, Dutta et al. evaluated grasp abilities by deploying intelligent algorithms with healthy subjects and post-stroke patients using an instrumented glove composed of six flex sensors, three force sensors, and a motion processing unit [17]. During the Wolf Motor Function Test execution, Del Din et al. used six accelerometers placed on the arm and the trunk to estimate Fugl–Meyer Assessment Test scores [18]. Finally, Routhier et al. studied the correlation between finger-to-nose task (FNT) and upper limb motor function in subacute stroke patients, using an IMU [19]. Although many of these studies evaluated the UEs with sensory information during OMs, none of them focused on the assessment of hand function by studying the range of motion (ROM) of the finger joints. Nevertheless, several studies have been conducted on the functional range of motion (FROM) of the finger joints during the performance of ADLs in healthy subjects [20,21,22]. Despite this, only Bain et al., who used the Sollerman hand grip function test [23], and Hayashi et al., who used 19 activities of the Disabilities of the Arm, Shoulder, and Hand (DASH) [24], used rehabilitation OMs. To the best of our knowledge, no study has determined the FROM and the ROM of the finger joints in stroke patients during the performance of the ARAT using a data glove. This study aimed to determine whether differences in the FROM and the ROM of finger joints between healthy subjects and post-stroke patients allow the identification of joint motion impairments and compensatory strategies in stroke patients that are not detected with the ARAT. The data obtained are of clinical importance for physiotherapists, as they allow a more quantitative and objective evaluation method.

## 2. Materials and Methods

### 2.1. Subjects

Twelve patients (3 women and 9 men, mean age: 65.2 ± 9.3 years; right-handed) were evaluated six months after a stroke at Sant Joan de Deu Hospital. Ten patients suffered an ischemic stroke, and two patients a hemorrhagic stroke. Inclusion criteria for this study included the following: patients who had a stroke for the first time with motor deficits in the UEs; patients older than 18 years; patients who, before the stroke, were independent in their ADLs; patients with a global ARAT score ≥ 10. Exclusion criteria: patients with UE deficits and sequelae of any etiology before the stroke. Data from the control group used in this study were taken from a publicly available dataset [25] obtained in previous research. The dataset includes information from 25 healthy subjects (14 women and 11 men, mean age: 40.2 ± 18.1 years). Inclusion criteria were being right-handed, over 18 years old, and not having suffered any hand disorders or injury. Healthy subjects performed sixteen activities of the ARAT corresponding to the subtests (Grasp, Grip, and Pinch) using an instrumented glove (Cyberglove Systems LLC; San Jose, CA, USA). All subjects signed informed consent to the protocol, which was conformed following the Declaration of Helsinki and was approved by the Ethics and Clinical Research Committee of the Fundacio Unió Catalana d’Hospitals ID 13/71. The stroke patients were divided into two groups to evaluate and detect impairments of the finger joints. Therefore, we formed one group of patients with hemiparesis on the right side and the other with hemiparesis on the left side. The information of the two stroke groups and the control group is shown in Table 1.

### 2.2. Experimental Protocol

In the present study, post-stroke patients performed sixteen tests (see Table 2) of the ARAT. These tests correspond to the Grasp, Grip, and Pinch subtests. The ARAT is an evaluative measure used to assess the arm motor status after a stroke, consisting of 19 tests categorized into four subtests: Grasp, Grip, Pinch, and Gross movements. Within each subtest, the first test is the most difficult and the second the easiest to facilitate the application of the test [9]. The Gross movement subtest was excluded because it involves the assessment of large muscle movements and, in this study, we focused on measuring the finger joints. Stroke patients sat upright in a standard chair with a firm back and no armrests. The assessments were performed in the hospital by a trained therapist. Subjects were seated in front of a table; the table was set at a distance of 15 cm and at the abdomen level. The physical therapist ensured that the subject’s back remained in contact with the back of the chair and that the legs were positioned in front of the chair with the feet in contact with the floor throughout the test. The subject was asked to grasp, lift vertically, place, and then release each object (block, cricket ball, or marble) onto the top of the shelf. The objects used in each activity were placed one at a time on the table. The ARAT performance score is rated on a 4-point scale, ranging from 0 (no movement) to 3 (movement performed normally). A full description of all ARAT tasks was presented in [10]. In this study, only the ARAT activities that the patient was able to complete, which obtained a score of 2 (complete task that takes a little longer) and 3 (complete task), were analyzed and compared with the control group.

### 2.3. Experimental Equipment

**Figure 1:**
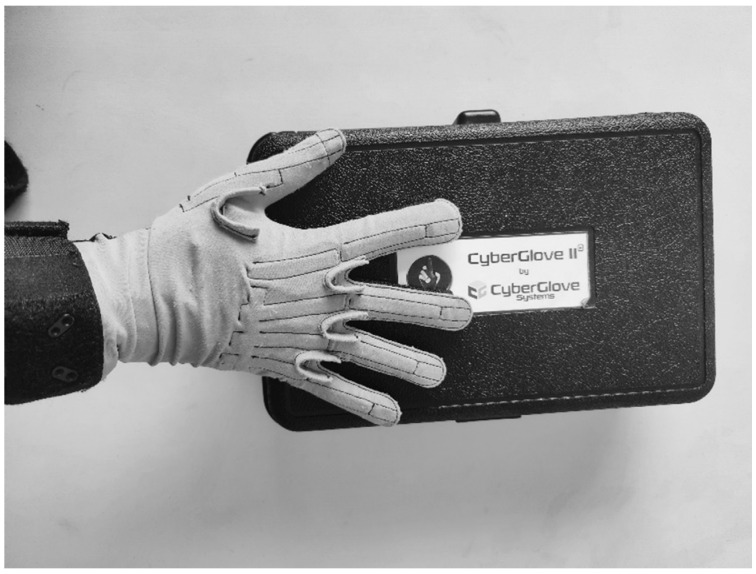
A participant wearing the CyberGlove II^®^.

Subjects performed the sixteen activities of the ARAT wearing the CyberGlove II^®^ data glove on the affected hand of subjects with hemiparesis and on the right hand (dominant) of healthy subjects (Figure 1). The data glove is composed of 18 flexion sensors: two bend sensors on each finger, four abduction sensors, and sensors measuring thumb crossover, palm arch, wrist flexion, and wrist abduction. The data glove has a resolution <1 degree and weighs only 70 g [26]. The procedure for converting the readings of the 18 sensors into finger joint angles was based on linear interpolation, according to a previously validated calibration protocol [27,28]. The eleven finger joints angles recorded in this study were: Thumb carpometacarpal (CMC) joint, Thumb, Index, Middle, Ring, and Little metacarpophalangeal (MCP) joints, Thumb interphalangeal (IP) joint, and Index, Middle, Ring, and Little proximal interphalangeal (PIP) joints. Data from the CyberGlove II^®^ were transmitted to a PC via Bluetooth connection. To read and record the data, a user interface (UI) was developed in Unity^®^ software (Unity Technologies Inc., San Francisco, CA, USA) version 2021.1.20. A script was created in order to convert the raw data of the CyberGlove II^®^ into angles according to the equations obtained in the calibration process. The UI allows visualizing the angle of the finger joints in real time, and evaluation of each activity. The angles of the eleven finger joints obtained in each test were recorded in a Comma-Separated Values (CSV) file for statistical analysis.

### 2.4. Data Analysis

The data obtained were filtered with a 2nd-order two-way low pass Butterworth filter with a cut-off frequency of 5 Hz in MATLAB^®^ software MathWorks, Inc., Natick, MA, USA. The following protocol was applied separately to the control group and the stroke groups. At the start of each test, the subject placed the hand tested pronated, immediately lateral to the testing object. Therefore, the initial instants of each record, in which the hand were static, were trimmed. The minimum and maximum values for each activity were calculated for each finger joint of each subject. The respective values were averaged across all subjects during each activity; these values became known as the extension and flexion angles (E/F). Then, the functional range of motion (FROM) was calculated as the 5th and 95th percentiles of the (E/F) angles of each finger joint in the sixteen activities, thus representing the maximum and minimum angles covering 90% of the activities at each specific finger joint. The FROM was used based on 90% of activities because considering 100% of activities may result in excessive values [21,23]. Alternatively, the range of motion (ROM) was defined as the average of the E/F angles of the finger joints during the sixteen activities of the ARAT. Similarly, the total arc of motion (aROM) was defined as the range of flexion and extension angles that compose the ROM. Finally, the range of motion for each finger joint in each subtest (sROM) was calculated. The sROM was defined as the average of the extension and flexion angles corresponding to the activities of the subtest considered.

Statistical analysis was conducted using IBM SPSS Statistics, Version 28.0. Armonk, NY, USA: IBM Corp. The respective extension and flexion angles (ROM) of each finger joint were compared between control and each stroke group using a non-parametric test, the Mann–Whitney U test. In each subtest (Grasp, Grip, and Pinch), the Mann–Whitney U test was used to compare whether there was a statistical difference in the sROM of the finger joints between the control group and each stroke group. Additionally, the flexion angles of the FROM in each finger joint were compared between the right hemiparesis, left hemiparesis, and control groups. For this purpose, a Welch’s ANOVA and a Games–Howell post hoc test was used to detect significant differences. Lastly, the ROM and aROM of each finger joint were compared between the right hemiparesis and the left hemiparesis groups using the Mann–Whitney U test. A *p*-value of less than 0.05 was considered statistically significant for all statistical analyses.

## 3. Results

### 3.1. Functional Range of Motion of the Finger Joints

**Figure 2:**
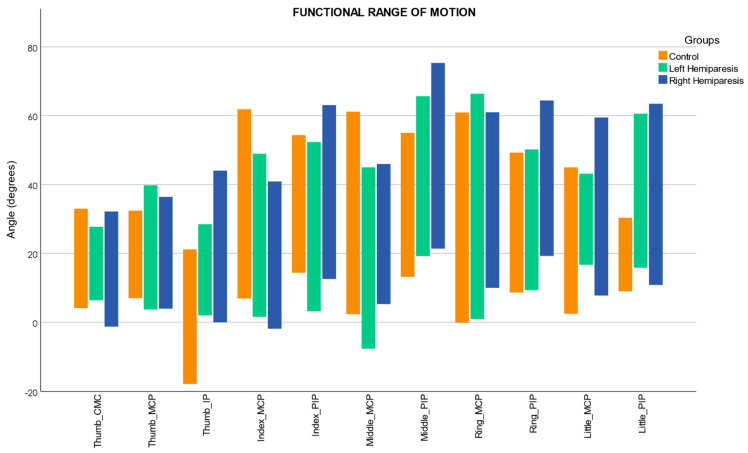
Functional range of motion in each finger joint; CMC = carpometacarpal; MCP = metacarpophalangeal; IP = interphalangeal; PIP = proximal interphalangeal; negative values represent hyperextension; maximum = flexion; minimum = extension.

The functional range of motion (FROM) of the finger joints required to perform 90% of the activities for each group is shown in Figure 2. In this study we decided to analyze the mean flexion angles of the 14 tasks that integrate the FROM. Mean and standard deviation values of the mean flexion angles (FROM) of each finger joint in the control, right hemiparesis (RH), and left hemiparesis (LH) groups are shown in Table 3. A Welch’s ANOVA revealed that there was a statistically significant difference in the flexion angle of the Thumb IP, Index MCP, Index PIP, Middle MCP, Middle PIP, Ring PIP, and Little PIP finger joints between the control, right hemiparesis (RH), and left hemiparesis (LH) groups. The results of the post hoc test (see Table 3) showed that the mean flexion angles of the Index MCP and Middle MCP in the control group were significantly larger than those in the RH group. In contrast, the mean flexion angles of the Thumb IP, Middle PIP, Ring PIP, Little MCP, and Little PIP in the RH group were significantly larger than those in the control group. The mean flexion angles of the Thumb IP, Middle PIP, and Little PIP in the LH group were significantly higher than those in the control group. However, the mean flexion angles in the Middle MCP joint in the control group were significantly larger than those in the RH group. Moreover, the mean flexion angles of the Thumb IP and Middle MCP in the LH group were significantly higher than those in the RH group. Lastly, the mean flexion angles of the PIP joints (Index, Middle, and Ring) in the RH group were significantly larger than those in the LH group.

### 3.2. Range of Motion of the Finger Joints in the Stroke Group with Right Hemiparesis

Mean and standard deviation values of the range of motion (ROM) and the total arc of motion (aROM) of each finger joint in the control and the stroke group with right hemiparesis (RH) are shown in Table 4. As reported in Table 5, the extension angles of the Thumb CMC, Index MCP, Middle MCP joints in the control group were significantly lower than those in the RH group. In contrast, the RH group showed significantly lower extension angles in the Thumb IP, Middle PIP, Ring MCP, Ring PIP, Little MCP, and Little PIP joints. The flexion angles of the Index MCP and Middle MCP joints in the control group were significantly higher than those in the RH group, while flexion angles of the Thumb MCP, Thumb IP, Index PIP, Middle PIP, Ring MCP, Ring PIP, Little MCP, and Little PIP joints in the RH group were significantly higher (see Table 5). The aROM in the control group was significantly larger than that in the RH group in the Middle MCP joint. By comparison, the aROM of the Thumb MCP, Index PIP, Middle PIP, Ring PIP, and Little PIP joints in the RH group was significantly larger (see Table 5).

### 3.3. Range of Motion of the Finger Joints in the Stroke Group with Left Hemiparesis

The range of motion (ROM) and the total arc of motion (aROM) of each finger joint in the control and the stroke group with left hemiparesis (LH) are shown in Table 6. As shown in Table 7, the extension angles of the Thumb CMC, Index MCP and PIP, Middle MCP joints in the control group were significantly lower than those in the LH group. In contrast, the LH group showed significantly lower extension angles in the Thumb IP, Middle PIP, Little MCP, and Little PIP joints. The flexion angles of the Index (MCP, PIP) and Middle MCP joints in the control group were significantly larger than those in the LH group, while flexion angles in the LH group were significantly larger in the Thumb MCP, Thumb IP, Middle PIP, Ring MCP, Little MCP, and Little PIP joints (see Table 7). The aROM of the Thumb IP and Little MCP joints in the control group was significantly larger than that in the LH group. In addition, aROM in the LH group was significantly larger in the Thumb MCP and Little PIP joints (see Table 7).

### 3.4. Comparison of the Range of Motion between the Stroke Groups

The results of the comparison between the stroke groups are shown in Table 8. The results showed than the extension angles of the Thumb CMC and Little (MCP, PIP) joints in the left hemiparesis (LH) group were significantly lower than those in the right hemiparesis (RH) group. In contrast, the extension angles of the Index PIP and Middle MCP joints in the RH group were significantly lower. The flexion angles of the PIP joints of the Index, Middle and Ring fingers in the RH group were significantly larger than those in the LH group, while, in the LH group, the flexion angle of the Index MCP joint was significantly larger. Lastly, the arc of motion of the Thumb IP, Middle PIP, Ring PIP and Little MCP in the LH group was significantly larger.

## 4. Discussion

To the best of our knowledge, there are no previous studies that measured and evaluated the finger joint motions during a standardized outcome measure such as the ARAT test. In this study, we determined the functional range of motion (FROM) and the range of motion (ROM) of the finger joints of the right hand, with the exception of distal interphalangeal (DIP) joints, using a data glove (CyberGlove II^®^) while performing the Grasp, Grip, and Pinch subtests of the ARAT. The study was conducted in healthy subjects and post-stroke subjects with a global ARAT score ≥ 10.

In this study, both the FROM and ROM were analyzed. The FROM is the amplitude of motion necessary for each finger joint to perform 90% of the activities of the ARAT (14 activities). The FROM has been used in several studies to determine the minimum range of motion needed to comfortably and effectively perform activities of daily living [20,22,24,29]. A table with the functional range of motion (FROM) for each finger joint is included in the Supplementary Material (Table S1). Determining the FROM allowed us to detect if there is a decrease in the arc of motion in some of the finger joints, and thus to establish rehabilitation therapy goals. To the best of our knowledge, there are no previous studies regarding the FROM in stroke patients. However, the FROM is highly dependent on the activities performed and is normally used with activities of daily living (ADLs) [21,22]. Therefore, we also decided to determine the ROM for a more in-depth evaluation since the ROM analyses relate to flexion and extension angles during the sixteen tests of the ARAT. The results of the flexion angles in the FROM (see Table 3) showed that the control group performed significantly greater flexion with the Index MCP and Middle MCP joints than the stroke groups, whereas no significant differences were found in the flexion angles at the Ring MCP joint. In contrast, the right hemiparesis (RH) group performed larger flexion angles in PIP joints of the Index, Middle, Ring, and Little fingers, whereas the left hemiparesis (LH) group performed larger flexion angles in the PIP joints of the Middle finger. The results in the RH stroke group suggest that they use a compensatory grasping strategy for the deficit of flexion in the Index MCP and Middle MCP joints. By comparison, the LH stroke group used a similar strategy for the deficit of flexion in the Middle MCP joint. In the ARAT, most of the activities are radial activities that include precision grip and pinch (Grip and Pinch subsets); as a result, the Index and Middle joints are essential in most of the tests.

In addition, the results of the ROM in the RH and LH stroke groups showed significantly larger extension angles (closer to 0 deg) in the Index MCP and Middle MCP joints than those in the control group. Finger joint extension problems may occur because, after stroke, the ability to extend the fingers during grip is highly variable due to issues with the active extensor muscles of the fingers and the coordination of muscle activity between the flexor and extensor muscles of the fingers [30]. In Carpinella et al., patients with hemiplegic stroke showed significantly lower extension and flexion angles than healthy subjects in all the finger joints (MCP, IP) during hand open and closing movements [31]. By comparison, in our study, the LH and RH stroke groups showed significantly lower flexion angles in the Index MCP and Middle MCP joints than healthy subjects. However, the RH stroke group showed significantly larger flexion in the Index, Middle, Ring, and Little PIP joints. Moreover, the LH stroke group showed significantly larger flexion in the Middle and Little PIP joints than in the control group. The difference with Carpinella et al. is that their study only evaluated hand movement (open and close). In contrast, our study assessed ROM of the finger joints during the performance of sixteen activities with different objects (shape and size).

A previous study found a relationship between the size of the object and the fingers used when grasping an object [32]. According to Peña-Pitarch et al., in the Grasp subtest, healthy subjects used five, four, or three fingers. In contrast, the subjects used a three-jaw chuck pinch, involving the pads of the thumb as opposed to the pads of the Index and Middle fingers in the Grip subtest. Activities in the Grasp subset involve power grasping. Power grasping is usually used when the object needs to be held firmly and involves the ulnar side of the hand. In contrast, in the Grip subtest, most activities include precision grasping, which is used to perform fine-grained actions that require accuracy [33]. In addition, the Grasp activities involve global activities where the radial and ulnar sides of the hand are employed. In our study, the RH patients showed significantly larger flexion angles in the Ring MCP, Little MCP, and the PIP joints than the control group in the Grasp and Grip subset (see Tables S2 and S3), but significantly lower flexion in the Index and Middle MCP joints. Nevertheless, RH patients in the Grip subset, which involves radial activities (precision grip and pinch), used greater flexion in the ulnar side of the hand. Furthermore, RH patients showed increased PIP joint flexion angles, indicating a compensatory strategy involving increased Index PIP and Middle PIP flexion as compensation for reduced flexion angles in the Index MCP and middle MCP joints. Furthermore, the LH patients showed significantly lower flexion angles in the Middle MCP in the Grasp and Grip subtest but significantly larger flexion angles in the Middle PIP in the grip subtests (see Tables S2 and S3). Therefore, the LH patients showed a PIP compensation strategy similar to the RH patients in the Middle joints. In addition, LH patients showed reduced flexion angles in the Index MCP and Index PIP joints. On the other hand, several studies [34,35] showed that, in a precision pinch, the Index finger worked actively and the Thumb worked passively, i.e., the Index joints performed a more significant flexion movement than the Thumb joints. Similar results were observed in the control group during the Pinch subset in our study. At the same time, RH patients showed impairment at the MCP joints of the Index and Middle fingers, compensating with increased flexion of the Thumb MCP and IP joints, and the PIP joints of the Index, Middle, and Ring fingers (see Table S4). Furthermore, the LH group in the Pinch subtest showed reduced flexion angles in the Index MCP, Index PIP, and Middle MCP, compensating with increased flexion of the Thumb CMC, Thumb MCP, Middle PIP, and Ring PIP joints. Therefore, during the Pinch subtest, the LH and RH stroke patients also used the PIP strategy to compensate for the flexion deficit in the MCP joints. In addition, we found the same compensation strategy during the FROM analysis (see Figure 2), showing that this metric performed reliably with the ARAT tasks.

Raghavan et al. found that stroke patients with right hemiparesis used a compensatory strategy that involved increased MCP flexion rather than the PIP flexion seen in controls [36]. The stroke patients showed reduced flexion angles at the PIP joints and extension angles at the MCP joints when grasping three objects of different shapes (rectangular, concave, and convex) wearing an instrumented glove. The difference with our study is that Raghavan et al. found the compensatory strategy in stroke patients with significant impairments, as were noted in their scores on the Fugl–Meyer Scale (FMS). In contrast, our study found the compensatory strategy in LH and RH stroke patients with moderate and good recovery, who obtained an ARAT global score greater than ten before the study. Furthermore, in Raghavan et al., stroke patients were evaluated during the grasping of only three objects, and in our study, stroke patients were assessed using 12 different objects. In addition, we found the compensatory strategy in the assessment of patients with left and right hemiparesis.

Finally, we evaluated patients with right and left hemiparesis separately in this study. Although patients with right-sided hemiparesis had the dominant hand affected, the results showed similar behavior in both groups. However, the results showed that, in the LH group, the flexion angle of the Index MCP joint was significantly higher than that in the RH group. In contrast, the index PIP joint flexion angle was larger in the RH group. Therefore, we determined that the LH group presented the compensatory strategy (PIP) in the Middle finger, whereas the RH group presented the PIP strategy in the Index and Middle fingers. Movement deficits in finger joints in patients with right hemiparesis found in our study suggested that patients with RH suffered a more severe stroke. Moreover, Hedna et al. found that left hemispheric ischemic strokes appear to be more frequent and have a worse outcome [37]. In our study, seven patients presented stroke in the left hemisphere and five in the right hemisphere. In addition, patients having suffered a stroke in the right hemisphere showed a higher ARAT score, consistent with that presented by Hedna et al. However, given the small and selected sample in this study, we are unable to generalize these compensatory strategies to post-stroke patients overall.

Importantly, the results presented in this study showed that the integration of the CyberGlove II^®^ during the performance of the ARAT allows for a more quantitative and sensitive assessment of post-stroke patients. In addition, analyzing and measuring the FROM and ROM of the finger joints revealed the compensatory strategies used for impairments in the finger joints of stroke patients. This research, however, is subject to several limitations. Firstly, the subjects in this study had a moderate and good recovery; futures studies should evaluate subjects with more severe impairments for more complete results. Secondly, it was impossible to obtain consent from the hospital’s ethics committee to have access to more stroke patients due to the restrictions of COVID-19 and the risk of SARS-CoV-2 infection among patients with a history of stroke. Thirdly, the method proposed in this study to evaluate the finger joints is not compatible with the Gross movement subtest of the ARAT because this test evaluates the movement of the arm. Finally, abduction and adduction angles of the finger joints were not obtained and not assessed in this study.

## 5. Conclusions

The results presented in this study demonstrated that the integration of a data glove (CyberGlove II^®^) during the performance of a validated clinical test such as the ARAT can be used to determine the range of motion (ROM) and the functional range of motion (FROM) of the finger joints. Therefore, the assessment of the FROM and ROM allowed us to detected finger joint impairments and compensatory grasp strategies in stroke patients that were not detected using clinical scores. The present study is of clinical relevance and allows for a more accurate and sensitive evaluation of a validated test, which would help physiotherapists and other health professionals to create rehabilitation programs focused on the recovery of hand function in stroke patients. However, future studies should consider a sample of more stroke subjects and incorporate an inertial sensor system to assess hand motion.

## Figures and Tables

**Table 1:** Characteristics of the groups.

Variable	Groups		
	RH	LH	C
Age (Mean ± SD)	62 ± 10.3	69.6 ± 5.3	40.2 ± 18.1
Hemisphere Affected	L	R	-
Subjects (N)	7	5	25
S. Grasp (tests)	31	24	150
S. Grip tests (tests)	19	16	100
S. Pinch tests (tests)	30	24	150
Total (tests)	80	64	400
TSS	6	6	-
ARAT score (Mean ± SD)	39.2 ± 14.3	45.4 ± 13.7	-

RH = right hemiparesis; LH = left hemiparesis; C = control group; SD = standard deviation; L = left; R = right; N = Number of participants; tests = complete test (ARAT score ≥ 2); S = subtest; TS = time since stroke (months).

**Table 2:** Description of the sixteen tests performed.

Subtest	Test	Description
Grasp	1	Grasp a block (10 cm^3^), lift vertically, place, and then release onto the top of the shelf.
	2	Grasp a block (2.5 cm^3^), lift vertically, place, and then release onto the top of the shelf.
	3	Grasp a block (5 cm^3^), lift vertically, place, and then release onto the top of the shelf.
	4	Grasp a block (7.5 cm^3^), lift vertically, place, and then release onto the top of the shelf.
	5	Grasp a cricket ball (diameter, 7 cm), lift vertically, place, and then release onto the top of the shelf.
	6	Grasp a sharpening stone (10.0 × 2.5 × 1 cm), lift vertically, place, and then release onto the top of the shelf.
Grip	7	Pour water from one glass to another.
	8	Displace alloy tube (diameter, 2.25 cm) from one side of table to the other.
	9	Displace alloy tube (diameter, 1 cm) from one side of table to the other.
	10	Put a washer over bolt (outer diameter, 3.5 cm; inner diameter,1.5 cm).
Pinch	11	Ball bearing (diameter, 6 mm), held between ring finger and thumb.
	12	Marble (diameter, 1.6 cm), held between index finger and thumb.
	13	Ball bearing (diameter, 6 mm), held between middle finger and thumb.
	14	Ball bearing (diameter, 6 mm), held between index finger and thumb.
	15	Marble (diameter, 1.6 cm), held between ring finger and thumb.
	16	Marble (diameter, 1.6 cm), held between middle finger and thumb.

**Table 3:** Flexion angles of the functional range of motion (FROM) during 14 tests.

Finger Joints	C		RH		L		M(C-RH)		M(C-LH)		M(RH-LH)	
	F	SD	F	SD	F	SD	M	*p*	M	*p*	M	*p*
Thumb CMC	28.2	5.0	26.5	4.8	26.6	0.7	1.65	0.629	1.55	0.474	−0.10	0.996
Thumb MCP	26.4	3.3	29.1	4.2	29.9	5.0	−2.70	0.143	−3.48	0.084	−0.78	0.887
Thumb IP	12.6	4.1	26.8	5.1	21.7	4.1	−14.21	0.000 ***	−9.16	0.000 ***	5.04	0.016 **
Index MCP	45.0	8.3	35.8	3.4	40.4	6.2	9.18	0.002 **	4.53	0.224	−4.64	0.047 *
Index PIP	37.8	8.7	43.7	11.3	30.6	11.4	−5.84	0.269	7.24	0.143	13.09	0.010 *
Middle MCP	46.4	7.0	34.9	4.6	34.8	7.0	11.46	0.000 ***	11.54	0.000 ***	0.083	0.999
Middle PIP	41.6	6.8	59.6	9.9	51.3	8.1	−17.99	0.000 ***	−9.77	0.003 **	8.22	0.048 *
Ring MCP	42.8	9.3	49.6	7.3	48.8	10.7	−6.78	0.085	−5.99	0.246	0.79	0.970
Ring PIP	38.2	6.5	54.2	7.3	39.2	6.9	−16.00	0.000 ***	−1.02	0.909	14.97	0.000 ***
Little MCP	30.1	6.6	38.9	9.9	33.4	5.1	−8.80	0.023 *	−3.34	0.284	5.45	0.166
Little PIP	21.3	4.2	47.2	11.5	48.1	8.8	−25.87	0.000 ***	−26.73	0.000 ***	−0.86	0.971

Games–Howell post-hoc comparison; C = control group; RH = right hemiparesis; LH = left hemiparesis; F = flexion; SD = standard deviation; CMC = carpometacarpal; MCP = metacarpophalangeal; IP = interphalangeal; PIP = proximal interphalangeal; negative values represent hyperextension; * *p* < 0.05; ** *p* < 0.01; *** *p* < 0.001; M = mean differences between groups; *p* = significance level.

**Table 4:** Range of motion (ROM) during the sixteen activities (control and right hemiparesis).

	Extension (Degree)				Flexion (Degree)				aROM (Degree)			
	C		RH		C		RH		C		RH	
Finger Joints	Mean	SD	Mean	SD	Mean	SD	Mean	SD	Mean	SD	Mean	SD
Thumb CMC	9.7	7.5	5.6	7.5	28.6	7.4	26.8	10.0	18.9	6.4	21.2	9.6
Thumb MCP	12.6	9.3	11.3	10.5	26.8	8.9	29.7	11.4	14.3	7.3	18.4	10.6
Thumb IP	−7.5	16.2	6.4	16.4	13.2	14.9	28.3	16.9	20.7	13.7	21.9	15.6
Index MCP	22.2	13.1	11.2	15.3	46.2	13.5	36.5	12.4	24.0	12.1	25.4	13.3
Index PIP	16.4	9.2	15.8	10.3	38.9	14.0	44.2	16.5	22.4	11.1	28.4	16.8
Middle MCP	17.8	11.9	14.0	12.8	47.5	11.5	36.0	12.6	29.7	10.9	22.0	10.2
Middle PIP	16.3	8.5	27.3	14.5	42.6	11.4	60.0	15.4	26.3	9.3	32.7	15.5
Ring MCP	15.6	11.8	20.6	14.7	44.1	13.6	49.8	13.0	28.5	11.4	29.3	15.7
Ring PIP	12.3	8.1	23.9	13.6	39.2	11.8	54.3	14.1	26.9	9.8	30.5	12.2
Little MCP	9.1	8.8	13.2	8.0	31.5	12.1	39.3	15.1	22.4	8.9	26.2	14.9
Little PIP	11.3	9.5	15.4	11.0	22.1	13.4	47.5	21.6	10.8	8.3	32.1	19.6

C = control group; RH = right hemiparesis group; CMC = carpometacarpal; MCP = metacarpophalangeal; IP = interphalangeal; PIP = proximal interphalangeal; SD = standard deviation; deg = degrees; aROM = arc of motion; negative values represent hyperextension.

**Table 5:** Results of Mann–Whitney test of the ROM with respect to the control and right hemiparesis groups.

			Extension				Flexion				aROM			
Finger Joint	Group	N	Mean Rank	U	Z	*p*	Mean Rank	U	Z	*p*	Mean Rank	U	Z	*p*
Thumb CMC	C	400	252.9	11,023.0	−4.39	0.000 ***	245.6	13,959.5	−1.80	0.072	236.2	14,262.0	−1.53	0.125
	RH	80	178.3				215.0				262.2			
Thumb MCP	C	400	243.1	14,949.0	−0.93	0.353	234.2	13,489.0	−2.22	0.027 *	231.8	12,518.0	−3.07	0.002 **
	RH	80	227.4				271.9				284.0			
Thumb IP	C	400	222.4	8764.5	−6.39	0.000 ***	219.1	7435.0	−7.56	0.000 ***	239.5	15,610.0	−0.34	0.731
	RH	80	330.9				347.6				245.4			
Index MCP	C	400	257.7	9136.0	−6.06	0.000 ***	256.6	9555.5	−5.69	0.000 ***	238.4	15,146.0	−0.75	0.451
	RH	80	154.7				159.9				251.2			
Index PIP	C	400	242.9	15,058.0	−0.83	0.406	232.7	12,877.5	−2.76	0.006 **	233.1	13,055.0	−2.60	0.009 **
	RH	80	228.7				279.5				277.3			
Middle MCP	C	400	248.6	12,758.0	−2.86	0.004 **	259.7	8302.5	−6.80	0.000 ***	258.2	8940.0	−6.23	0.000 ***
	RH	80	200.0				144.3				152.3			
Middle PIP	C	400	223.0	9002.0	−6.18	0.000 ***	215.7	6092.5	−8.75	0.000 ***	230.6	12,029.0	−3.51	0.000 ***
	RH	80	328.0				364.3				290.1			
Ring MCP	C	400	231.6	12,459.0	−3.13	0.002 **	229.8	11,706.0	−3.79	0.000 ***	240.8	15,874.0	−0.11	0.911
	RH	80	284.8				294.2				238.9			
Ring PIP	C	400	220.7	8085.0	−6.99	0.000 ***	217.0	6593.5	−8.31	0.000 ***	233.9	13,363.0	−2.33	0.020 **
	RH	80	339.4				358.1				273.5			
Little MCP	C	400	230.4	11,941.0	−3.58	0.000 ***	228.0	11,012.0	−4.40	0.000 ***	236.8	14,521.0	−1.31	0.192
	RH	80	291.2				302.9				259.0			
Little PIP	C	400	231.4	12,372.0	−3.20	0.001 **	213.5	5192.0	−9.54	0.000 ***	211.6	4450.0	−10.20	0.000 ***
	RH	80	285.9				375.6				384.9			

C = control group; RH = right hemiparesis group; * *p* < 0.05; ** *p* < 0.01; *** *p* < 0.001; control vs. stroke Mann–Whitney U test.; N = number of tests per group.

**Table 6:** Range of motion (ROM) during the sixteen activities (control and left hemiparesis).

Finger Joints	Extension (Deg)				Flexion (Deg)				aROM (Deg)			
	C		LH		C		LH		C		LH	
	Mean	SD	Mean	SD	Mean	SD	Mean	SD	Mean	SD	Mean	SD
Thumb CMC	9.7	7.5	8.1	1.8	28.6	7.4	26.7	1.5	18.9	6.4	18.6	1.6
Thumb MCP	12.6	9.3	11.1	6.5	26.8	8.9	30.4	10.0	14.3	7.3	19.2	10.9
Thumb IP	−7.5	16.2	7.2	8.7	13.2	14.9	22.7	8.5	20.7	13.7	15.5	9.4
Index MCP	22.2	13.1	14.0	18.9	46.2	13.5	41.8	13.0	24.0	12.1	27.9	21.9
Index PIP	16.4	9.2	8.4	11.9	38.9	14.0	32.5	19.3	22.4	11.1	24.1	15.2
Middle MCP	17.8	11.9	7.1	13.1	47.5	11.5	36.4	15.5	29.7	10.9	29.3	18.6
Middle PIP	16.3	8.5	25.8	11.4	42.6	11.4	52.2	13.1	26.3	9.3	26.3	11.8
Ring MCP	15.6	11.8	17.4	13.4	44.1	13.6	50.9	17.3	28.5	11.4	33.5	16.7
Ring PIP	12.3	8.1	15.8	10.0	39.2	11.8	40.1	13.2	26.9	9.8	24.4	11.8
Little MCP	9.1	8.8	21.1	4.6	31.5	12.1	34.5	7.3	22.4	8.9	13.4	7.3
Little PIP	11.3	9.5	21.7	10.4	22.1	13.4	49.6	16.9	10.8	8.3	27.8	15.9

C = control group; LH = left hemiparesis group; CMC = carpometacarpal; MCP = metacarpophalangeal; IP = interphalangeal; PIP = proximal interphalangeal; SD = standard deviation; deg = degrees; aROM = arc of motion; negative values represent hyperextension.

**Table 7:** Results of Mann–Whitney test of the ROM with respect to the control and left hemiparesis groups.

			Extension				Flexion				aROM			
Finger Joint	Group	N	Mean Rank	U	Z	*p*	Mean Rank	U	Z	*p*	Mean Rank	U	Z	*p*
Thumb CMC	C	400	239.04	10,184	−2.63	0.009 **	241.31	9276.5	−3.54	0.000 ***	231.11	12,245	−0.56	0.577
	LH	64	191.63				177.45				241.17			
Thumb MCP	C	400	236.86	11,055	−1.75	0.080	225.95	10,181	−2.63	0.009 **	223.10	9038	−3.78	0.000 ***
	LH	64	205.23				273.42				291.28			
Thumb IP	C	400	214.15	5459	−7.37	0.000 ***	216.74	6494.5	−6.33	0.000 ***	239.12	10,152	−2.66	0.008 **
	LH	64	347.20				331.02				191.13			
Index MCP	C	400	239.85	9862	−2.95	0.003 **	237.69	10,724	−2.08	0.037 *	232.16	12,665	−0.14	0.892
	LH	64	186.59				200.06				234.61			
Index PIP	C	400	246.80	7081	−5.74	0.000 ***	240.10	9761	−3.05	0.002 **	232.36	12,744	−0.06	0.955
	LH	64	143.14				185.02				233.38			
Middle MCP	C	400	248.15	6540	−6.29	0.000 ***	244.94	7825	−5.00	0.000 ***	236.83	11,070	−1.74	0.082
	LH	64	134.69				154.77				205.47			
Middle PIP	C	400	216.99	6595	−6.23	0.000 ***	219.62	7649	−5.17	0.000 ***	234.35	12,061	−0.74	0.458
	LH	64	329.45				312.98				220.95			
Ring MCP	C	400	229.43	11,573	−1.23	0.218	224.78	9712.5	−3.10	0.002 **	227.78	10,910	−1.90	0.058
	LH	64	251.67				280.74				262.03			
Ring PIP	C	400	227.95	10,981	−1.83	0.068	230.32	11,926	−0.88	0.380	237.09	10,963	−1.84	0.065
	LH	64	260.92				246.16				203.80			
Little MCP	C	400	207.59	2836	−10.00	0.000 ***	224.78	9712	−3.10	0.002 **	251.12	5353	−7.48	0.000 ***
	LH	64	388.19				280.75				116.14			
Little PIP	C	400	216.00	6198	−6.63	0.000 ***	207.51	2805	−10.04	0.000 ***	211.14	4257	−8.58	0.000 ***
	LH	64	335.66				388.67				365.98			

C = control group; LH = left hemiparesis group; * *p* < 0.05; ** *p* < 0.01; *** *p* < 0.001; control vs. LH Mann–Whitney U test.; N = number of tests per group.

**Table 8:** Results of Mann–Whitney test of the ROM with respect to the stroke groups (left hemiparesis vs. right hemiparesis).

			Extension				Flexion				aROM			
Finger Joint	Group	N	Mean Rank	U	Z	*p*	Mean Rank	U	Z	*p*	Mean Rank	U	Z	*p*
Thumb CMC	LH	64	82.45	1923.5	−2.56	0.010 **	72.65	2550.5	−0.04	0.970	69.84	2390	−0.68	0.494
	RH	80	64.54				72.38				74.63			
Thumb MCP	LH	64	70.77	2449.5	−0.44	0.657	73.89	2471	−0.36	0.720	75.74	2352.5	−0.83	0.404
	RH	80	73.88				71.39				69.91			
Thumb IP	LH	64	74.30	2445	−0.46	0.644	66.08	2149	−1.65	0.098	61.88	1880	−2.73	0.006 **
	RH	80	71.06				77.64				81.00			
Index MCP	LH	64	76.67	2293	−1.08	0.282	82.09	1946	−2.47	0.013 **	71.86	2519	−0.17	0.869
	RH	80	69.16				64.83				73.01			
Index PIP	LH	64	55.27	1457	−4.44	0.000 ***	57.24	1583.5	−3.93	0.000 ***	66.38	2168	−1.58	0.115
	RH	80	86.29				84.71				77.40			
Middle MCP	LH	64	60.95	1820.5	−2.97	0.003 **	73.53	2494	−0.27	0.791	78.67	2165	−1.59	0.112
	RH	80	81.74				71.68				67.56			
Middle PIP	LH	64	70.84	2454	−0.43	0.670	59.94	1756	−3.23	0.001 **	61.84	1878	−2.74	0.006 **
	RH	80	73.83				82.55				81.03			
Ring MCP	LH	64	66.91	2202.5	−1.44	0.151	73.13	2519.5	−0.16	0.871	78.56	2172	−1.56	0.119
	RH	80	76.97				71.99				67.65			
Ring PIP	LH	64	57.68	1611.5	−3.81	0.000	51.55	1219.5	−5.39	0.000 ***	61.54	1858.5	−2.82	0.005 **
	RH	80	84.36				89.26				81.27			
Little MCP	LH	64	96.03	1054	−6.06	0.000 ***	66.45	2173	−1.56	0.120	50.23	1135	−5.73	0.000 ***
	RH	80	53.68				77.34				90.31			
Little PIP	LH	64	85.30	1741	−3.29	0.001 **	76.23	2321	−0.96	0.337	68.06	2276	−1.14	0.253
	RH	80	62.26				69.51				76.05			

RH = right hemiparesis group; LH = left hemiparesis group; C = control group; S = stroke group; * *p* < 0.05; ** *p* < 0.01; *** *p* < 0.001; control vs. stroke Mann–Whitney U test.; N = number of tests per group.

## Data Availability

The datasets generated and analyzed during the current study are available at this repository Padilla, Jesus (2022): Finger Joints Angles ARAT. Figshare. Dataset. https://doi.org/10.6084/m9.figshare.19467269.v1 (accessed on 4 March 2022).

## Supplementary Materials

The following supporting information can be downloaded at: https://www.mdpi.com/article/10.3390/s22103604/s1. Table S1. Functional Range of Motion (FROM) for each finger joint; Table S2: Subtest Range of Motion (sROM) during the performance of the Grasp subtest (All groups); Table S3: Subtest Range of Motion (sROM) during the performance of the Grip subtest (All groups); Table S4: Subtest Range of Motion (sROM) during the performance of the Pinch subtest (All groups).
