# Supplementary material for: Quantitative Assessment of Hand Function in Healthy Subjects and Post-Stroke Patients with the Action Research Arm Test
Source: Sensors (Basel). 2022 May 10;22(10):3604. doi: 10.3390/s22103604 (PMC9147783; doi:10.3390/s22103604)
Supplement: Supplementary file 1 [file sensors-22-03604-s001.zip › sensors-1683277-supplementary.pdf]

sensors

Supplementary Materials

Quantitative Assessment of Hand Function in Healthy Subjects and Post-Stroke Patients with the Action Research Arm Test

Jesus Fernando Padilla-Magaña [1,2,*], Esteban Peña-Pitarch [1], Isahi Sánchez-Suarez [2] and Neus Ticó-Falguera [3]

[1] Escola Politècnica Superior d'Enginyeria de Manresa (EPSEM), Polytechnic University of Catalonia (UPC), 08242 Manresa, Spain; esteban.pena@upc.edu
[2] Department of Manufacturing Technologies, Polytechnic University of Uruapan Michoacán, 60210 Uruapan ,Michoacán, Mexico; i.sanchez@upu.edu.mx
[3] Physical Medicine and Rehabilitation Service, Althaia Xarxa Assistencial de Manresa, 08243 Manresa, Spain; nico@althaia.cat
* Correspondence: jesus.fernando.padilla@upc.edu; Tel.: +34-671251375

Table S1. Functional Range of Motion (FROM) for each finger joint

Finger Joints	Groups		
	C	LH	RH
Thumb CMC	4.09 - 32.97	6.36 - 27.68	-1.25 - 32.19
Thumb MCP	6.99 - 32.40	3.70 - 39.78	3.92 - 36.4
Thumb IP	-17.82 - 21.17	1.96 - 28.48	-0.05 - 44.04
Index MCP	6.90 - 61.89	1.53 - 48.93	-1.84 - 40.92
Index PIP	14.36 - 54.36	3.18 - 52.36	12.55 - 63.1
Middle MCP	2.32 - 61.17	-7.65 - 45	5.27 - 45.94
Middle PIP	13.17 - 55.02	19.19 - 65.67	21.39 - 75.34
Ring MCP	-0.14 - 60.92	0.90 - 66.38	10 - 61.01
Ring PIP	8.62 - 49.25	9.31 - 50.21	19.24 - 64.42
Little MCP	2.44 - 44.97	16.68 - 43.17	7.74 - 59.47
Little PIP	8.96 - 30.34	15.82 - 60.60	10.83 - 63.46

C= control group; RH= right hemiparesis; LH= left hemiparesis; CMC= carpometacarpal; MCP = metacarpophalangeal; IP = interphalangeal; PIP = proximal interphalangeal; Negative values represent hyperextension; FROM represents the amplitude of the motion.

Table S2. Subtest Range of Motion (sROM) during the performance of the Grasp Subtest (All groups)

Finger Joints	C (°)		LH (°)		RH (°)		C (°)		LH (°)		RH (°)	
	E	SD	E	SD	E	SD	F	SD	F	SD	F	SD
Thumb CMC	5.9	7.4	6.7	1.0	2.0**	7.8	24.0	7.6	26.1	1.7	22.2	9.1
Thumb MCP	10.4	9.5	9.7	5.6	9.3	10.3	24.3	8.8	26.9	10.3	26.8	12.0
Thumb IP	-2.6	14.3	7.3***	6.8	6.6**	18.1	13.3	11.9	22.2***	7.2	32.5***	21.1
Index MCP	17.7	15.8	3.8***	18.4	4.7***	17.0	42.7	11.0	41.6	13.9	36.5**	12.3
Index PIP	15.6	8.9	5.0***	11.4	13.8	8.6	35.2	10.6	24.6***	17.3	38.6	13.8
Middle MCP	11.9	12.9	-2.0***	12.3	8.4	12.5	43.3	9.9	35.8**	16.5	32.3***	12.0
Middle PIP	15.9	7.4	20.4	8.3	24.8***	9.8	41.7	9.0	45.6	12.5	53.1***	12.3
Ring MCP	7.9	11.6	8.0	12.5	13.5	13.2	35.7	8.9	45.0**	17.0	43.3**	13.9
Ring PIP	12.3	8.1	10.2	3.9	21.4***	10.9	38.5	9.9	34.0	10.9	48.8***	13.4
Little MCP	7.3	7.4	17.8***	4.6	10.1	8.4	28.8	9.1	31.3	5.6	36.0**	14.5
Little PIP	11.8	9.7	18.6***	8.2	15.0	9.0	22.9	12.7	45.8***	16.2	44.0***	23.3

RH= right hemiparesis group; LH= left hemiparesis group; C= control group; E= extension; F= flexion; SD= standard deviation *p < 0.05; **p < 0.01; ***p < 0.001; Mann–Whitney U test; °=degrees; CMC= carpometacarpal; MCP = metacarpophalangeal; IP = interphalangeal; PIP = proximal interphalangeal;

Table S3. Subtest Functional Range of Motion (sROM) during the performance of the Grip Subtest (All groups)

Finger Joints	Control		LH		RH		Control		LH		RH	
	E	SD	E	SD	E	SD	F	SD	F	SD	F	SD
Thumb CMC	9.9	6.9	8.6	1.5	8.8	4.2	30.7	6.0	27.0*	1.2	28.1	7.6
Thumb MCP	10.6	8.6	9.0	6.1	8.2	8.8	28.4	9.5	29.8	9.6	28.7	9.8
Thumb IP	-11.6	17.4	4.5***	7.7	3.9***	12.9	18.6	17.9	25.3*	7.9	30.6***	9.7
Index MCP	21.4	11.0	14.1	17.8	11.9***	13.3	47.8	13.7	41.3	15.2	33.6***	10.2
Index PIP	18.4	10.1	12.2*	13.6	17.7	12.0	49.9	12.3	48.4	20.4	56.1	14.0
Middle MCP	17.5	8.7	8.2***	8.0	14.1*	10.6	48.7	11.8	36.2*	18.1	33.2***	10.1
Middle PIP	19.6	9.5	29.7**	14.5	30.3**	15.9	51.9	10.0	61.1**	10.8	67.5***	12.3
Ring MCP	16.6	9.4	19.4	11.2	23.2*	9.8	50.7	15.3	60.4	20.2	54.8	11.7
Ring PIP	14.1	7.9	18.4	12.3	25.0**	14.7	46.0	11.0	46.5	11.6	57.0**	15.6
Little MCP	12.5	7.4	22.4***	2.7	16.1*	7.2	41.7	12.9	40.6	7.3	51.2*	17.4
Little PIP	11.2	9.7	21.1**	12.4	13.3	13.7	25.2	14.4	53.8***	17.7	49.7***	24.1

RH= right hemiparesis group; LH= left hemiparesis group; C= control group; E= extension; F= flexion; SD= standard deviation *p < 0.05; **p < 0.01; ***p < 0.001; Mann–Whitney U test; °=degrees; CMC= carpometacarpal; MCP = metacarpophalangeal; IP = interphalangeal; PIP = proximal interphalangeal;

Table S4. Subtest Functional Range of Motion (sROM) during the performance of the Pinch Subtest (All groups)

Finger Joints	Control		LH		RH		Control		LH		RH	
	E	SD	E	SD	E	SD	F	SD	F	SD	F	SD
Thumb CMC	13.3	6.1	9.2***	1.6	7.2***	7.6	31.7	5.5	27.2***	1.2	30.7	10.6
Thumb MCP	16.0	8.6	14.0	6.8	15.4	10.8	28.3	8.0	34.2**	9.1	33.2	11.2
Thumb IP	-9.6	16.1	8.9***	10.6	7.6***	16.8	9.5	14.4	21.6	10.1	22.4***	13.9
Index MCP	27.1	9.3	24.1	14.8	17.3***	12.1	48.5	14.9	42.5**	11.1	38.5***	13.8
Index PIP	16.0	8.8	9.2***	10.7	16.6	10.9	35.3	14.4	29.9	14.3	42.2*	17.2
Middle MCP	24.0	9.3	15.6***	10.6	19.8	12.2	50.9	11.5	37.2***	13.3	41.7**	13.0
Middle PIP	14.6	8.2	28.7**	9.9	27.9***	17.5	37.4	10.7	52.7***	11.8	62.3***	17.4
Ring MCP	22.6	8.6	25.5	9.6	26.2*	16.0	48.1	12.0	50.4	12.9	53.4**	10.0
Ring PIP	11.2	8.0	19.6***	10.3	25.8***	15.4	35.4	12.1	41.9**	14.3	58.4***	12.1
Little MCP	8.7	10.3	23.5***	3.8	14.5**	7.3	27.4	10.1	33.6	6.4	35.3***	9.9
Little PIP	10.9	9.2	25.3***	10.3	17.2**	11.0	19.2	13.0	50.5	17.1	49.8***	18.1

RH= right hemiparesis group; LH= left hemiparesis group; C= control group; E= extension; F= flexion; SD= standard deviation *p < 0.05; **p < 0.01; ***p < 0.001; Mann–Whitney U test; °=degrees; CMC= carpometacarpal; MCP = metacarpophalangeal; IP = interphalangeal; PIP = proximal interphalangeal;
